# Dual targeting negative enrichment strategy for highly sensitive and purity detection of CTCs

**DOI:** 10.3389/fchem.2024.1400988

**Published:** 2024-05-20

**Authors:** Siying Gao, Xuejie Li, Zhiyuan Hu, Zihua Wang, Xiaopeng Hao

**Affiliations:** ^1^ School of Chemical Engineering and Pharmacy, Wuhan Institute of Technology, Wuhan, China; ^2^ CAS Key Laboratory for Biomedical Effects of Nanomaterials and Nanosafety, CAS Key Laboratory of Standardization and Measurement for Nanotechnology, CAS Center for Excellence in Nanoscience, National Center for Nanoscience and Technology, Beijing, China; ^3^ Fujian Provincial Key Laboratory of Brain Aging and Neurodegenerative Diseases, School of Basic Medical Sciences, Fujian Medical University, Fuzhou, China; ^4^ School of Nanoscience and Technology, SinoDanish College, University of Chinese Academy of Sciences, Beijing, China; ^5^ Department of General Surgery, First Medical Center of Chinese PLA General Hospital, Beijing, China

**Keywords:** circulating tumor cells (CTCs), CD45, CD66b, enrichment methods, clinical detection

## Abstract

Circulating tumor cells (CTCs) have significant clinical value in early tumor detection, dynamic monitoring and immunotherapy. CTC detection stands out as a leading non-invasive approach for tumor diagnostics and therapeutics. However, the high heterogeneity of CTCs and the occurrence of epithelial-mesenchymal transition (EMT) during metastasis pose challenges to methods relying on EpCAM-positive enrichment. To address these limitations, a method based on negative enrichment of CTCs using specific leukocyte targets has been developed. In this study, aiming to overcome the low purity associated with immunomagnetic beads targeting solely the leukocyte common antigen CD45, we introduced CD66b-modified immunomagnetic beads. CD66b, a specific target for neutrophils with abundant residues, was chosen as a complementary approach. The process involved initial collection of nucleated cells from whole blood samples using density gradient centrifugation. Subsequently, magnetically labeled leukocytes were removed by magnetic field, enabling the capture of CTCs with higher sensitivity and purity while retaining their activity. Finally, we selected 20 clinical blood samples from patients with various cancers to validate the effectiveness of this strategy, providing a new generalized tool for the clinical detection of CTCs.

## 1 Introduction

Cancer remains a significant disease that seriously threaten the health of lives worldwide. A pivotal characteristic that distinguishes malignant tumors from benign ones is the formidable metastatic capability of the former, leading to the spread to distant organs and frequently culminating in patient fatality (Theil et al., 2022). CTCs, originating from malignant tissue, traverse into the peripheral blood circulation and disseminate to distant tissues (McGranahan and Swanton, 2017). CTC, thus, emerges as a valuable liquid biopsy biomarker with multifaceted applications in early cancer detection (Lawrence et al., 2023), diagnosis (Gazzaniga et al., 2014; Xu et al., 2020; Deng et al., 2022), prognosis (Jin et al., 2019; Pantel et al., 2019; Jin et al., 2023), monitoring (Punnoose et al., 2012; Castro-Giner and Aceto, 2020), and treatment (Yu et al., 2014; Praharaj et al., 2018; Alix-Panabières and Pantel, 2021).

Nevertheless, the isolation and enrichment of circulating tumor cells confront formidable challenges due to their rarity and heterogeneity (Balic et al., 2012). Despite decades of research and exploration, numerous methods for CTCs enrichment have been developed, primarily falling into two main categories based on separation principles: immunoaffinity-based strategies (Gorges et al., 2012) and those relying on biophysical properties such as cell size (Gabriel et al., 2016) and density (Vona et al., 2000; Pinzani et al., 2006; De Giorgi et al., 2010). Regarding separation techniques, immunomagnetic beads (Swennenhuis et al., 2016) and microfluidic microarrays (Kamande et al., 2013) constitute the two principal categories. The CellSearch system, founded on epithelial cell adhesion (EpCAM) immunomagnetic bead-positive enrichment of CTCs, has gained approval from the Food and Drug Administration (FDA) for clinical studies in breast, colorectal, and prostate cancers (Hong and Zu, 2013; Wang et al., 2016). However, methods relying on EpCAM for CTCs detection face challenges due to the heterogeneity of CTCs and the occurrence of epithelial-mesenchymal transition, resulting in the absence or downregulation of EpCAM expression on the cell surface (Zhang et al., 2013; Liao and Yang, 2020). Despite efforts to enhance capture efficiency using highly specific nucleic acid aptamers (Chen et al., 2019) and peptides (Dong et al., 2019), these bioactive molecules are susceptible to degradation and inactivation, limiting their clinical applicability.

In response to these limitations, several improvements have been proposed, including the introduction of mesenchymal markers such as N-cadherin and EGFR, as well as the utilization of bis-antibody-modified immunomagnetic beads for the isolation of CTCs(Wang et al., 2019). Moreover, the surface modification of magnetic nanobeads with biological acids like tannic acid (TA) has been explored. TA, binding to CTCs through the robust force of phenolic hydroxyl groups with the glycocalyx on cancer cell surfaces, exhibits a notable anti-leukocyte adhesion effect, reducing interactions with non-target cells (Ding et al., 2021). To address non-specific adsorption of background cells, such as leukocytes in blood, a method involving the modification of leukocyte membranes and neutrophil membranes onto immunomagnetic beads has been developed, effectively reducing non-specific protein adsorption (Zhang et al., 2019). However, most of these methods are intricate in preparation and cumbersome in operation. Consequently, there arises a pressing need for the development of a widely applicable and user-friendly CTCs isolation method.

In this study, we present a dual-antibodies nanomagnetic bead-based separation method designed for cancer patients and EpCAM-independent CTCs detection. Initially, red blood cells and platelets were eliminated from blood through density gradient centrifugation. Subsequently, magnetic beads with antibodies targeting leukocyte and neutrophils-specific antigens CD45 and CD66b were introduced to bind with these cells, facilitating their separation and removal under the magnetic field.

To optimize the recovery of CTCs, we systematically determined the most effective method for the preparation of dual antibody magnetic beads. The most suitable experimental conditions were determined by comparing different nanobeads concentrations, incubation times and other experiments. The method’s specificity and broad applicability were demonstrated using different breast cancer cell lines and cancer cells with *in vitro* induced epithelial-mesenchymal transition phenotypes, admixing them into the blood of healthy individuals to mimic cancer patient blood samples.

The sensitivity of our method was further validated through spiking experiments involving varying numbers of cancer cells. Notably, the recovered cancer cells exhibited high activity and retained their proliferative and growth capacities. Finally, we assessed the clinical applicability of the method in blood samples from 20 cancer patients and 10 healthy volunteers. The results underscore the universal utility of our approach as a versatile tool for the clinical detection of CTCs.

## 2 Materials and methods

### 2.1 Materials

Streptavidin-modified magnetic beads (200 nm) were purchased from Ademtech (France). Biotin-labeled CD45 antibody and biotin-labeled CD66b antibody was obtained from Biolegend (United States). FITC-labeled anti-EpCAM antibody, FITC-labeled anti-E-calmodulin antibody, APC-labeled anti-N-calmodulin antibody and Alexa Fluor 647-labeled anti-CD45 antibody were from Biolegend (United States). Alexa Fluor 488-labeled anti-CK antibody was purchased from Abcam (United States). Hoechst 33342 was from Beyotime (China). The human breast cell line (MDA-MB-468,MDA-MB-453,MDA-MB-231,MCF7) were from the Cell Resource Center, China Academy of Medical Sciences (China).

### 2.2 Synthesis and characterization of dual-antibody nanomagnetic beads

Biotin-labeled antibodies were ligated to streptavidin-modified magnetic beads via streptavidin-biotin interactions. The loading amount of the magnetic beads was 1 mg of streptavidin magnetic beads loaded with up to 10 µg of biotinylated antibody. Based on this, 20 μL and 10 µL of streptavidin-conjugated magnetic beads (5 mg/mL) were added to two 1.5 mL centrifuge tubes, respectively, and 2 µL and 1 µL of biotinylated CD45 antibody (0.5 mg/mL) and CD66b antibody (0.5 mg/mL) were added, respectively, and then PBS was added to the volume of the reaction system to be 100 µL and then incubated for 1 h.

The particle size distribution and zeta potential of CDmix@MNPs were tested by dynamic light scattering instrument (DLS,Nano-ZS-2019, Malvern, United Kingdom). The spectral properties of CDmix@MNPs were examined using a UV-Vis spectrophotometer (Lambda-950, Perkin Elmer Instruments, United States of America). The morphology of CDmix@MNPs was characterized by transmission electron microscopy (TEM, 120 kV, Hitachi-HT7700). The morphology of CDmix@MNPs captured leukocytes was characterized by scanning electron microscopy (SEM, WangZX-JSM-IT500). It was further verified by Prussian blue staining kit (Solarbio).

For SEM sample preparation, the wafers were first cleaned and dried with anhydrous ethanol, respectively. After coating the wafers with poly-lysine solution for 1 h, the magnetic beads and captured cells were added and settled for 1 h. The samples were dehydrated with 10%, 30%, 50%, 70%, 90%, and 100% ethanol for 10 min.

### 2.3 Cell culture and TGF-β induction

To induce the transformation of MCF7 cells from epithelial to mesenchymal phenotype MCF7 cells were starved with DMEM without fetal bovine serum for 24 h, and then 10 ng/mL of transforming growth factor-β (TGF-β) was added to DMEM containing 3% fetal bovine serum at 6 h, 24 h, and 36 h, respectively.

### 2.4 Spiked cell capture assay

To facilitate cell imaging and counting, cultured cells were pre-stained with Hoechst33342 staining solution after digestion with 0.25% trypsin in a cell incubator at 37°C. CDmix@MNPs were incubated with 5,000 cells for spiking cell capture experiments. The final captured recovered cells were counted by fluorescence microscope scanning.

### 2.5 Isolation of CTCs from cancer patients blood samples

Blood samples from 20 cancer patients and 10 healthy volunteers were obtained from clinical hospital. Peripheral blood samples were collected and used for CTC analysis. Informed consent was obtained from patients and healthy volunteers before blood collection. The study was approved by the Ethics Committee. Peripheral blood was collected using EDTA vacuum tubes, and blood samples were stored at 4°C and processed within 72 h after blood collection. Mononuclear cells were enriched from 1 mL of blood by density gradient centrifugation, and the supernatant was recovered by enrichment in a magnetic field after incubation with a quantitative amount of mixed magnetic beads, and the final cell precipitate was obtained by centrifugation at 1500 rpm for 10 min. Cells were incubated with Alexa Fluor 488-labeled anti-CK antibody and Alexa Fluor 647-labeled anti-CD45 antibody for 1 h and settled on slides. The slides were sealed using DAPI-containing sealer, and cells that were CK-positive, CD45-negative and had intact nucleus were counted by microscopy.

### 2.6 Statistics analysis

Statistical analyses were performed using GraphPad Prism (GraphPad Software, San Diego, United States). Differences with a *p*-value <0.05 were statistically significant.

## 3 Results

### 3.1 Characterization of dual-antibody magnetic beads

**FIGURE 1:**
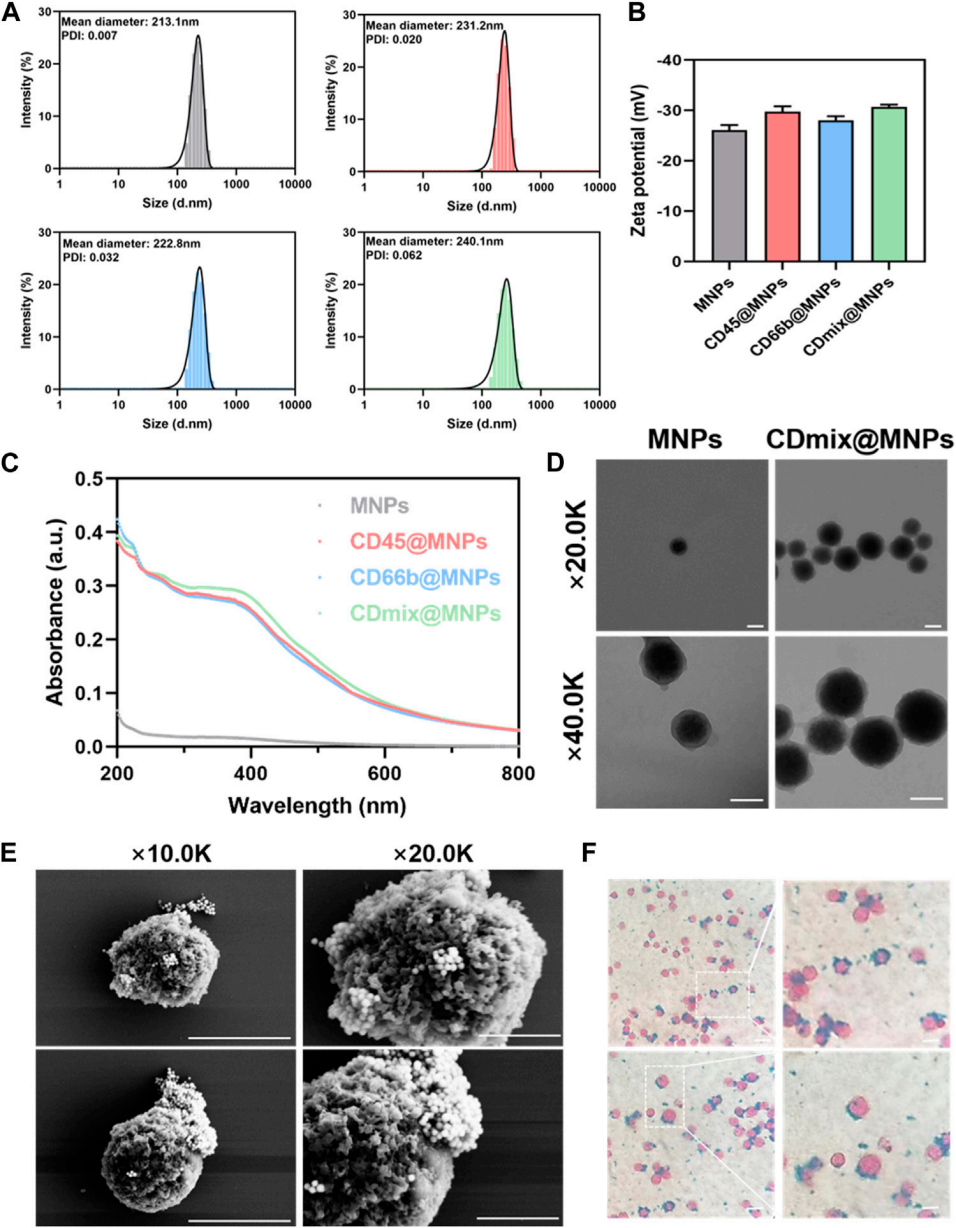
Characterization of CDmix@MNPs. **(A)** Size distribution curves of MNPs(grey), CD45@MNPs(red), CD66b@MNPs(blue) and CDmix@MNPs(green). **(B)** Zeta potentials of MNPs, CD45@MNPs, CD66b@MNPs and CDmix@MNPs. **(C)** UV-vis spectrophotometer graphs. **(D)** Morphology by TEM of MNPs and CDmix@MNPs (scale bar, 200 nm). **(E)** SEM images of leukocytes captured by CDmix@MNPs (scale bar, 5μm and 2 μm). **(F)** Prussian blue-stained images of leukocytes captured by CDmix@MNPs (scale bar, 10 μm).

The results from dynamic light scattering revealed the average sizes of MNPs, CD45@MNPs, CD66b@MNPs, and CDmix@MNPs to be 213.1 nm, 231.2 nm, 222.8 nm, and 240.1 nm, respectively (Figure 1A). Correspondingly, the zeta potentials were measured as −26.07 ± 1.02 mV, −29.77 ± 1.05 mV, −28.03 ± 0.81 mV, and −30.7 ± 0.44 mV, respectively (Figure 1B). UV-vis spectrophotometer graphs demonstrated that MNPs lacked characteristic absorption peaks within the studied wavelength range of 200–800 nm. In contrast, CD45@MNPs and CD66b@MNPs exhibited distinctive absorption peaks around 260 nm, while CDmix@MNPs displayed a more pronounced absorption peak at the same position (Figure 1C). Transmission electron microscopy was employed to investigate the topographic characteristics of MNPs and CDmix@MNPs. The results illustrated that the size of CDmix@MNPs was larger compared with that of bare MNPs, which confirmed the effective attachment of antibodies to the bare magnetic beads (Figure 1D). Further investigation of the morphology of CDmix@MNPs capturing leukocytes, conducted through scanning electron microscopy, revealed a substantial number of CDmix@MNPs adhering to the surface of leukocytes, underscoring the strong affinity between CDmix@MNPs and leukocytes (Figure 1E). Prussian blue staining provided additional evidence that CDmix@MNPs specifically recognized and targeted leukocytes, predominantly distributing on the cell surface (Figure 1F).

### 3.2 Design and optimization of capture methods

**FIGURE 2:**
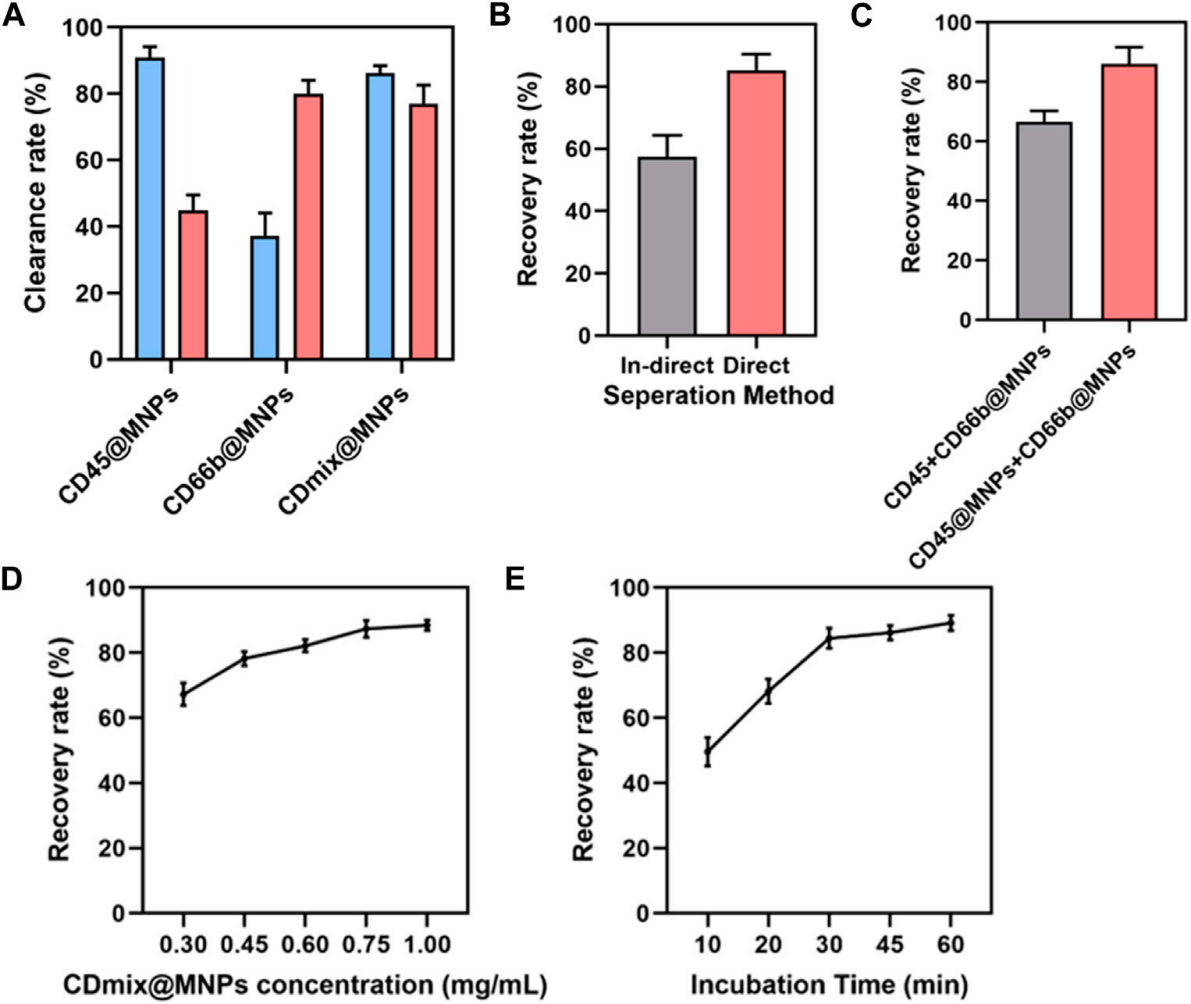
Design and optimization of capture methods. **(A)** Clearance of leukocytes and neutrophils from 1 mL of peripheral blood by three antibody magnetic beads. **(B)** Comparing the efficiency of direct and indirect methods for isolating cancer cells. **(C)** Comparison of two combinations of antibodies to magnetic beads. **(D)** The effect of hybrid magnetic beads concentrations on cancer cell recovery rate. **(E)** The effect of incubation time on the recovery rate of cancer cells.

Upon capturing 1 mL of peripheral blood using single CD45 antibody magnetic beads, the clearance of leukocytes exceeded 90%, yet the clearance of neutrophils was approximately 45%. Subsequent magnetic bead capture using CD66b antibody led to an increased neutrophil clearance of 80%, but the clearance of leukocytes dropped to less than 40%. Thus, a combination of CD45 and CD66b antibody beads became imperative. Dual-antibody hybrid bead capture achieved a leukocyte clearance exceeding 95%, while the neutrophil clearance rate approached 80% (Figure 2A). After establishing the initial capture method, further optimizations were undertaken. The effectiveness of direct and indirect methods for cancer cell recovery was compared. In the indirect method, cells were incubated with antibodies before the addition of magnetic beads, whereas in the direct method, antibodies were coupled with magnetic beads before incubation with cells. The direct method exhibited an approximate 20% increase in the recovery of cancer cells compared to the indirect method (*p* < 0.01) (Figure 2B). Subsequently, two methods of connecting the two antibodies to the magnetic beads were compared: mixing the antibodies first and then coupling them to the magnetic beads, or coupling the antibodies separately to the magnetic beads and then mixing. The latter method of attachment proved to be more effective (*p* < 0.01) (Figure 2C). The concentration of hybrid magnetic beads and incubation time were further studied to optimize capture conditions. The concentration of hybrid magnetic beads demonstrated a dual effect, with higher concentrations resulting in increased antibody attachment and faster separation. However, excessive concentrations led to self-aggregation of beads and increased non-specific binding. An optimal concentration of 0.75 mg/mL for hybrid magnetic beads was identified, as further increases did not significantly improve the cancer cells recovery rate (Figure 2D). The optimization of incubation time revealed that cancer cell recovery increased with longer incubation times, with 30 min identified as the optimal incubation time to meet the demand for rapid analysis (Figure 2E).

### 3.3 Dual antibody magnetic beads recovery of cancer cells

**FIGURE 3:**
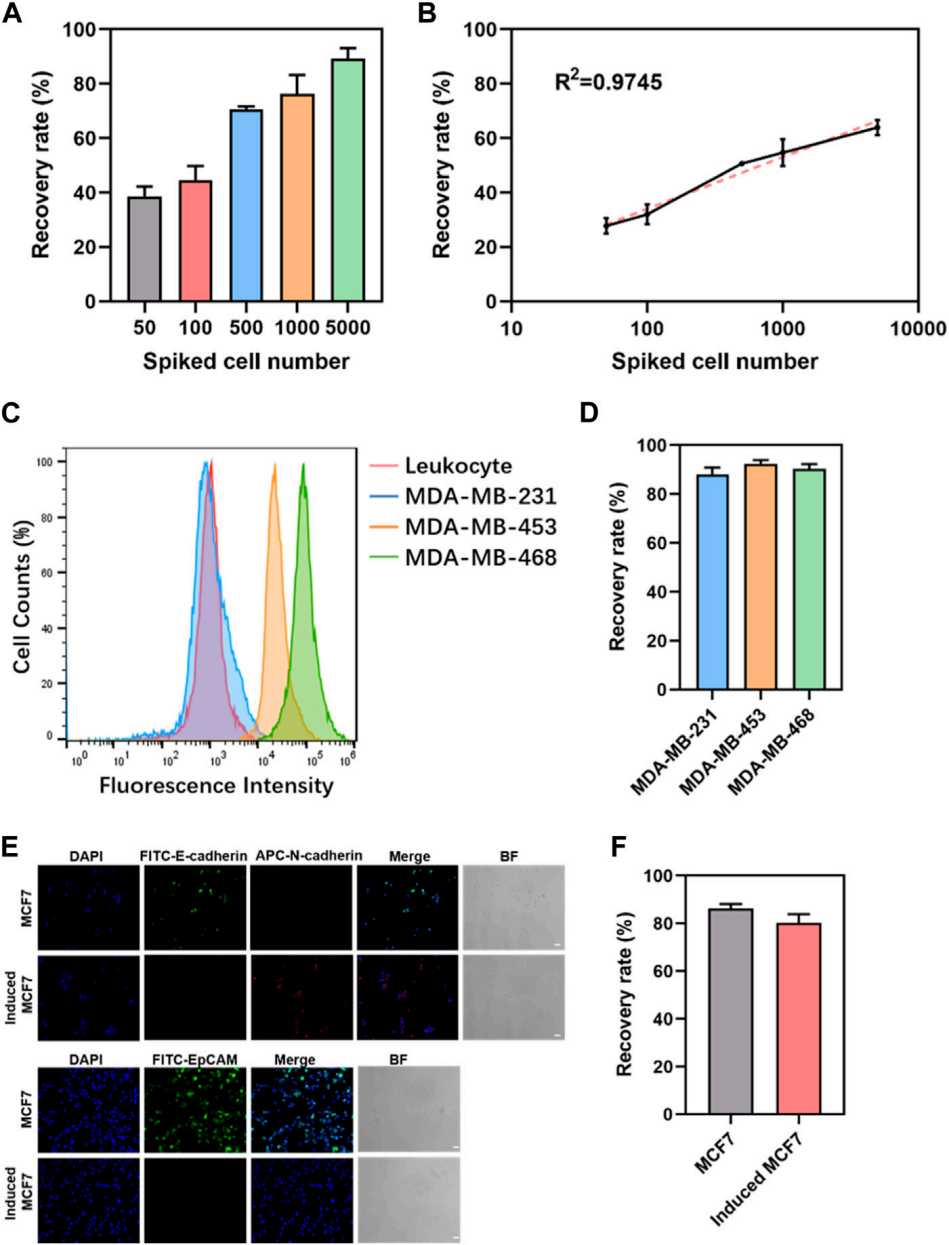
Sensitivity and generalizability of dual-antibody magnetic beads. **(A)** Recovery rate of different numbers of cancer cells spiked in 1 mL of peripheral blood. **(B)** Linear analysis of the recovery rate of cancer cells in 1 mL of peripheral blood admixed with different numbers of cancer cells. **(C)** EpCAM expression in leukocytes and three breast cancer cells. **(D)** Recovery rate of 5000 breast cancer cells spiked in 1 mL of peripheral blood. **(E)** Immunofluorescence staining graphs of MCF7 cells and induced MCF7cells (scale bar, 50 μm). **(F)** Recovery rate of 5000 MCF7 cells and induced MCF7 cells in 1 mL of peripheral blood.

The results indicate the successful optimization of capture conditions for dual antibody hybrid magnetic beads, demonstrating their efficacy in recovering spiked cancer cells (MDA-MB-468) at varying concentrations (50–5000/mL). The precision of spiked cancer cell counts was ensured through meticulous enumeration using fluorescence microscopy before their introduction into healthy blood samples. Notably, the dual antibody magnetic beads exhibited the capability to recover cancer cells even at a low concentration of 50 spiked cancer cells per 1 mL of blood sample, with a remarkable recovery rate exceeding 80% at a spiked concentration of 5000 cells/mL (Figure 3A). Linear analysis revealed a robust and positive linear relationship between the recovery rate of cancer cells and the number of spiked cancer cells, where recovery increased linearly with the number of spiked cells (Figure 3B).

Limitation of EpCAM-based positive enrichment due to phenotypic heterogeneity of CTCs and variability in EpCAM expression, three breast cancer cell lines with differing EpCAM expressions—MDA-MB-231, MDA-MB-453, and MDA-MB-468—were selected. Flow cytometry confirmed high, moderate, and minimal EpCAM expressions in MDA-MB-468, MDA-MB-453, and MDA-MB-231, respectively (Figure 3C). Spiking these breast cancer cell lines into 1 mL of peripheral blood mononuclear cells (PBMC) in peripheral blood showed no significant differences in recovery rates, regardless of EpCAM expression levels (Figure 3D). The robustness of this approach was further validated by inducing epithelial-mesenchymal transition in cancer cells using TGF-β. MCF7, a breast cancer cell line, exhibited significant downregulation of EpCAM and E-cadherin expression and upregulation of N-cadherin expression following TGF-β induction (Figure 3E). When 5000 MCF7 cells and induced MCF7 cells were admixed with 1 mL of peripheral blood for capture, no significant difference in recovery between the two groups was observed (Figure 3F). These findings collectively reinforce the versatility and robust performance of the dual antibody hybrid magnetic bead approach in capturing CTCs, even in the context of phenotypic variability and EMT induction.

### 3.4 Cell viability and proliferation

**FIGURE 4:**
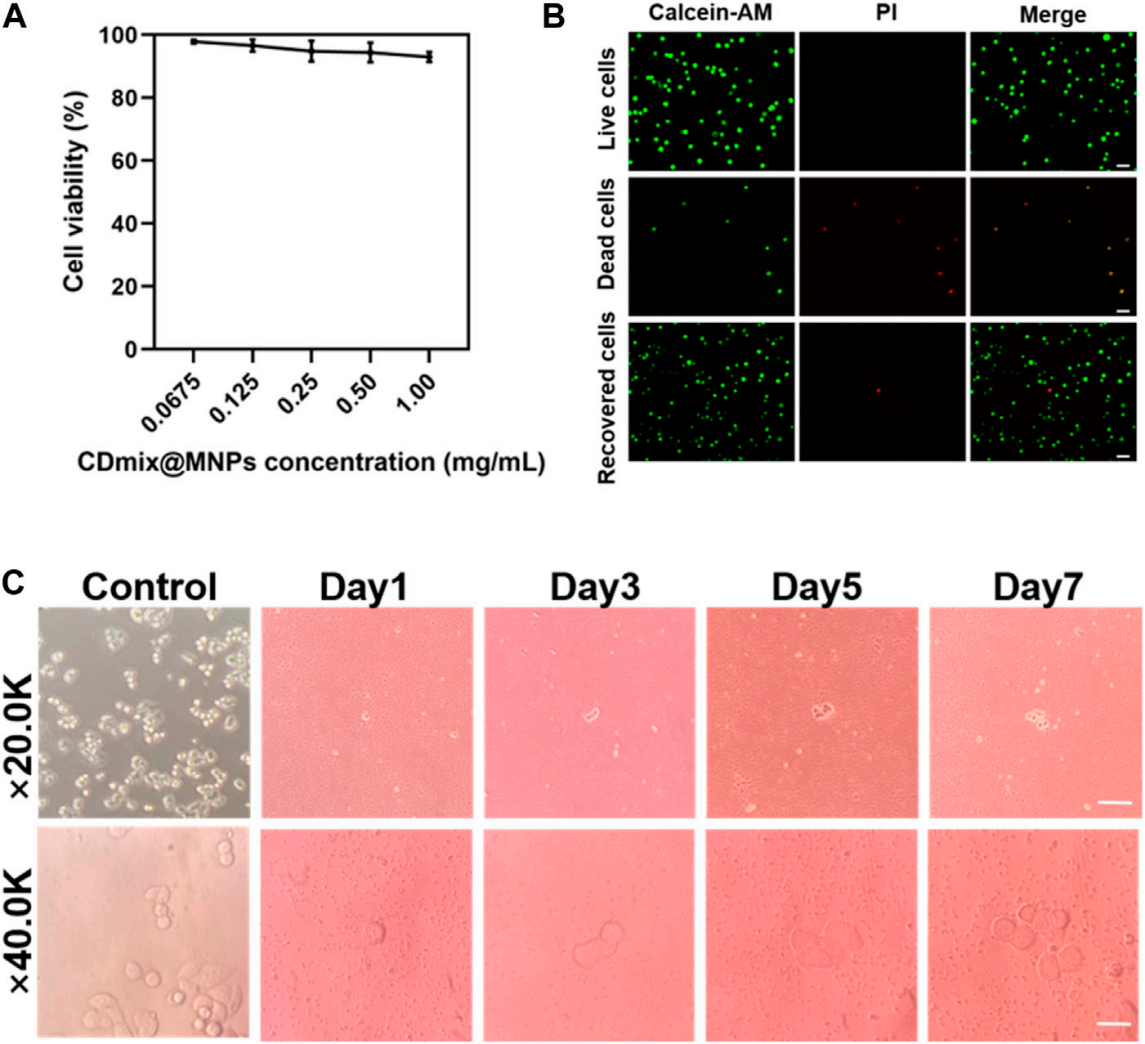
Viability and proliferation of recovered cancer cells. **(A)** MTT assay to measure the activity of captured cells. **(B)** Calcein-AM/PI staining of captured cells (scale bar, 20 μm). **(C)** Proliferation of cancer cells after negative enrichment for seven consecutive days (scale bar, 10 μm).

The impact of varying concentrations of hybrid magnetic beads on cell viability was investigated using the MTT method. The results demonstrated that as the concentration of hybrid magnetic beads increased from 0.0675 mg/mL to 1.00 mg/mL, there was no significant effect on cell viability during the 30 min incubation (Figure 4A). This indicates that the hybrid magnetic beads exhibit low toxicity to cells. To further assess the viability of recovered cells, Calcein-AM and propidium iodide (PI) dyes were employed for staining. Live and dead cell groups were included as controls, revealing that the vast majority of recovered cancer cells were viable (Figure 4B). This finding reinforces the notion that the hybrid magnetic beads exhibit good biocompatibility and are suitable for downstream analysis. Furthermore, the ability of recovered cancer cells to continue proliferating is a crucial aspect for subsequent functional analyses. Continuous culture of the recovered cancer cells for 7 days revealed that they maintained robust proliferative properties within this timeframe (Figure 4C). This observation underscores the suitability of the hybrid magnetic bead approach for downstream functional analyses, affirming the potential of this method in facilitating follow-up studies on recovered cancer cells.

### 3.5 CTCs in clinical samples

**FIGURE 5:**
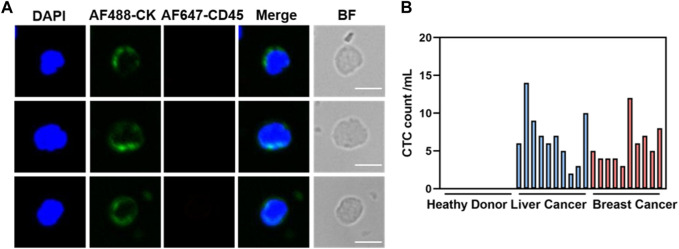
Clinical blood samples detected for CTCs. **(A)** Representative fluorogram of CTCs isolation from blood samples of 20 cancer patients using CDmix@MNPs (scale bar, 10 μm). **(B)** Number of CTCs isolated from 1 mL blood samples from 10 healthy donors and 20 cancer patients.

Given the remarkable cancer cell capture efficacy demonstrated by the hybrid magnetic beads *in vitro*, samples from cancer patients (n = 20) with various tumor types, including liver (n = 10) and breast (n = 10), as well as samples from healthy volunteers (n = 10), were collected for circulating tumor cell capture and enumeration. Peripheral blood (1 mL) underwent pretreatment via density gradient centrifugation to aspirate the peripheral blood mononuclear cell, which was then incubated with the optimal concentration of hybrid magnetic beads under optimal conditions. Immunofluorescence staining was employed to identify recovered samples after capture, with nuclei stained using DAPI, tumor cells and leukocytes differentiated using anti-cytokeratin (CK) and anti-CD45 antibodies. Cells that stained positively for DAPI and CK and negatively for CD45 were identified as cancer cells. Representative CK-positive and CD45-negative CTCs were observed in blood samples from all 20 cancer patients (Figure 5A), with no CTCs detected in blood samples from any of the 10 healthy volunteers. In contrast, 2–14 CTCs were isolated from hepatocellular carcinoma patient’s blood samples, and 4–12 CTCs were isolated from breast cancer patient’s blood samples (Figure 5B). The ability to completely differentiate between blood samples from cancer patients and healthy volunteers indicates that the capture of CTCs by dual antibody magnetic beads exhibits excellent specificity.

## 4 Discussion

In summary, we have designed and optimized a simple and versatile method for the negative enrichment of CTCs using dual-antibody-modified nanomagnetic beads. The CDmix@MNPs exhibit excellent recognition and removal capabilities for leukocytes and neutrophils, mitigating interference from background signals in samples. This versatility enables the recovery of various cancer cell types with different EpCAM expressions. Analysis of the CTCs detection results of clinical blood samples revealed that this method successfully distinguishes patient samples from those of healthy volunteers, minimizing the leakage rate of CTCs in clinical blood samples. This approach holds promise for advancing liquid biopsy techniques and improving the clinical management of cancer patients.

The identification of circulating tumor cells as a validated liquid biopsy cancer biomarker has opened avenues for real-time monitoring, yet their detection in peripheral blood poses significant challenges due to their rarity. Numerous enrichment techniques have been developed to isolate CTCs based on the biological or physical properties of tumor cells. Analyzing CTCs for mutations in specific molecular targets and biomarkers from cancer patient blood samples can predict responses to targeted therapies, facilitating precise and individualized cancer treatment (Ying, 2023). Additionally, the establishment of *in vitro* tumor models is instrumental in translating basic tumor research into clinical applications, playing a crucial role in understanding tumor pathogenesis and developing effective anti-tumor drugs. The isolation and amplification of cultured CTCs from various treatment stages offer insights into the dynamic evolution of tumor characteristics, guiding therapeutic decisions (Xu et al., 2023).

However, a limitation of our study is that, despite the universality of the isolation technique for different CTC subpopulations, we did not classify and analyze these subpopulations at the molecular and clinical levels. This information could contribute to more refined clinical applications and increased clinical value. Understanding the dynamic changes in CTC subpopulations, considering their different phenotypes, is crucial for predicting the prognosis and efficacy of cancer patients undergoing treatment. We believe that in the subsequent studies, we can combine the CTCs enrichment technique more closely with the downstream application analysis.

## Data Availability

The raw data supporting the conclusion of this article will be made available by the authors, without undue reservation.
